# When Women with Cystic Fibrosis Become Mothers: Psychosocial Impact and Adjustments

**DOI:** 10.1155/2016/9458980

**Published:** 2016-11-23

**Authors:** Sophie L. Cammidge, Alistair J. A. Duff, Gary J. Latchford, Christine Etherington

**Affiliations:** ^1^Leeds Institute of Health Sciences, University of Leeds, Charles Thackrah Building, 101 Clarendon Road, Leeds, West Yorkshire LS2 9LJ, UK; ^2^Regional Adult CF Unit, Leeds Teaching Hospitals NHS Trust, St James' University Hospital, Beckett Street, Leeds LS9 7TF, UK; ^3^Regional Paediatric CF Unit, Leeds General Infirmary, Great George Street, Leeds LS1 3EX, UK

## Abstract

Advances in the treatment and life expectancy of cystic fibrosis (CF) patients mean that motherhood is now a realistic option for many women with CF. This qualitative study explored the psychosocial impact and adjustments made when women with CF become mothers. Women with CF (*n* = 11) were recruited via an online forum and participated in semistructured telephone interviews about their experiences of becoming a mother. Transcriptions were analysed using Grounded Theory. Analysis revealed three core categories: (i) “Living with CF”: how becoming a mother impacted on health and treatment adherence, requiring a change in support from the CF team, (ii) “Becoming a Mother”: balancing issues common to new mothers with their CF, and (iii) “Pooling Personal Resources”: coping strategies in managing the dual demands of child and CF care. Participants experienced a variety of complex psychosocial processes. Most participants acknowledged an initial negative impact on CF care; however over time they reported successful adaptation to managing dual commitments and that adherence and motivation to stay well had improved. This study highlights the need for preconceptual psychosocial counselling and* postpartum* adjustment to CF care.

## 1. Introduction

Cystic fibrosis (CF) is a chronic life limiting condition. Adults with CF experience a build-up of mucus and bacteria in the lungs which is inefficiently cleared, leading to increased susceptibility to infection and respiratory problems. The digestive system is also affected and complications with liver functioning, diabetes, and osteoporosis are common.

Advances in care over the last 50 years have resulted in a life expectancy reaching the late 30s [1]. However, CF remains a progressive disease, with the most common cause of morbidity and mortality being respiratory failure. Treatment is time-consuming, complex, and arduous. A typical regimen includes the following: chest clearance physiotherapy, enzyme and nutritional supplementation, oral and inhaled antibiotics and mucolytics, and recommendations for daily exercise. Care also involves regular outpatient appointments and when necessary, inpatient stays for intravenous antibiotic treatment. Patients must also adhere to strict infection control guidelines which involve avoiding contact with other patients and known environmental and domestic risks (e.g., hay and Jacuzzis).

With increased survival, the issues of pregnancy and motherhood have become even more important. Many adults with CF have expectations of healthy sexual relationships and hope for children [2], and, with pregnancy and motherhood being realistic prospects for women with CF, many are becoming parents [3].

Studies have highlighted the significant, and often difficult, psychosocial impact of parenthood for any woman and the coping and adjustment processes that take place [4–9]. However, it has been argued that the stresses and pressures associated with “healthy” motherhood may be heightened in mothers with a chronic illness. Research suggests that chronically ill mothers do indeed experience a variety of complex psychological and emotional processes [10–20].

While women with CF can successfully conceive and carry a pregnancy [21, 22], some studies have shown they have reduced lung function in the two years* postpartum* [3, 23] although others have found that this can return to baseline within six months [24]. Such work is plagued with a lack of control groups meaning findings are equivocal.

To date there has been little exploration of the psychosocial impact of motherhood on women with CF [25]. This is in spite of it being well known that information is crucial in informing preconceptual counselling and* postpartum* psychosocial support [22]. Due to the desire for, and significant lack of, information in this area, this research aimed to develop an understanding of the psychosocial issues mothers with CF experience and their processes of coping and adjustment. The results will enable CF teams to provide more detailed information to women with CF on the psychosocial issues associated with motherhood as and when appropriate. This will enable teams to support women with CF in making decisions about having children, in planning and preparing for motherhood, and in raising awareness on the psychosocial support a woman may require on becoming a mother. To the best of our knowledge, this study is the first to qualitatively explore the psychosocial experiences of mothers with CF and how they cope and adjust.

## 2. Methods

### 2.1. Ethics

Ethical approval was obtained from the University of Leeds (HSLTLM/11/011). All participants gave fully informed written consent. Pseudonyms have been used throughout the manuscript.

### 2.2. Design

We utilised a qualitative methodology, Grounded Theory (GT), which enabled detailed exploration of experience. GT is a widely used, inductive method of analysis, designed to develop theory through rigorous and credible identification and integration of categories of data. Categories or themes are identified from transcripts and, as analysis progresses, categories at increasingly higher, analytic levels of abstraction are identified. Emergent “theory” is therefore “grounded” in the data [26]. Our study strictly adhered to the procedures for implementation of GT [27]. Service-user consultation was incorporated into all research-design stages.

### 2.3. Participants and Recruitment

Eleven mothers with CF were recruited from an online forum (CFMothers.com), which was established for women with CF who are interested in having, or have had, children, in order to share information and experiences. Our study was advertised on the forum and interested participants contacted the lead investigator (first author), whereupon initial contact by e-mail was made to arrange the postage and signing of consent forms, completion of quantitative measures, and interview times.

Table 1 details participants' health and treatment status. Median forced expiratory volume in one-second percentage (FEV_1_, a measure of lung function) was 71% (range 27–103) and median body mass index (BMI) was 22 (range 20–27). This is comparable to CF Registry data [1].

### 2.4. Data Collection

Qualitative data was generated from semistructured telephone interviews (mean length 57 minutes) which, commensurate with GT principles [27], were adapted following each interview to further explore emerging questions and concepts. The decision was made to conduct telephone interviews for a number of reasons. Telephone interviews enabled recruitment of participants from across the UK. They also allowed participants full flexibility in terms of the timing of interviews. It was also felt that face-to-face interviews may make it harder for those mothers who were more unwell, or who may struggle to arrange child care, to take part. It was felt that participants may wish to speak about difficult or sensitive issues and that they would feel more able to do this over the telephone. Indeed, participants in psychological research have been shown to be more likely to give socially desirable answers in face-to-face interviews than during telephone ones [28]. Therefore, we felt that richer information would be gathered from telephone interviews. Interviews were recorded and professionally transcribed verbatim and then cross-checked with the original recordings by the lead investigator.

The Hospital Anxiety and Depression Scale (HADS) [29] and CF Quality of Life (CFQoL) Scale [30] were also administered to screen for specific difficulties with emotional functioning and quality of life.

### 2.5. Analysis

GT involves three levels of analysis: open, axial, and selective coding. Additional processes and “analytic tools” to enhance rigour are also utilised (e.g., “constant comparison,” where all new codes are compared to all existing codes to ensure consistency and differentiation). Analysis and collection ran concurrently.

Open coding involves analysing transcripts “line by line” and assigning “open codes” to each, designed to capture the essence of the content. Each interview yielded between 160 and 270 open codes that were then grouped into “conceptual categories” related in meaning. Between 12 and 18 conceptual categories emerged from each interview.

Axial coding involves the development of subcategories in order to enhance the explanatory power of the conceptual categories. Using open codes as a “starting point,” constant comparison of the data within and between conceptual categories led to their refinement. Between 39 and 79 subcategories emerged from each interview.

Selective coding involves reviewing previous categories in light of new ones generated from the most recent interview. This enabled validation (or disconfirmation) of new and existing categories contributing to the emerging overall model. The conceptual categories and subcategories emerging from the most recent interview were then integrated into the previous analysis, with relationships between categories being noted and the new categories helping to refine the overall model.

Final analysis yielded 15 conceptual categories and 175 subcategories. Here selective coding, which also involves integration and refinement of theory through the identification of core categories, took precedence. At this point, the GT model was also refined. Excess categories (i.e., which only one participant had contributed to) were excluded and similar subcategories collapsed and relabelled.

On completion of GT modelling there was one central process, three core categories, 13 conceptual categories, and 70 subcategories. This paper focuses on describing the 13 conceptual categories.

### 2.6. Quality Standards in Qualitative Research

Several methods for assuring quality were adhered to [27, 31, 32], including supervision of the first author by the third author throughout analysis and discussion of coding choices. Credibility checks were also completed. This involved the first author sending a copy of the results (via e-mail) to all participants. This included a copy and explanation of the theoretical formulation and a description of all the categories included. Participants were asked to comment on the extent to which the results concurred with their experiences. All participants consented to being contacted for this purpose at the initial interview stage. Eight out of the 11 participants replied and all agreed that the categories captured and reflected their experiences of being a mother. Feedback included the following:

“*I loved reading the results. They've really captured my experiences of being a mother. I honestly can't think of anything that you have missed.*” (Tamsin)

*“I think they are an accurate description of what motherhood and CF involves. It will be very helpful for other women with CF wanting to try for a baby, and give a real insight into how other women cope. I wish I had had this information when I became pregnant.”* (Kimberley)

*“I found them really interesting to read and quite comforting in that I was surprised how many others must share my feelings, difficulties etc. There were also some points I didn't say but on reading them felt a real affinity with.”* (Joanna)

A “verification step” was also completed, which involved the first author reviewing the data for discrepancies and overstatements in the final stages of analysis. Finally, an auditor check was conducted. This involved a colleague, with experience of conducting qualitative research at Ph.D. level and who was familiar with GT, checking the categories derived from the analysis. In order to do this he was provided with one randomly selected page from each of the 11 transcripts, with the originally selected meaning units and their allocated open codes marked on the page (total of 50). He was also provided with a list of the final core categories, conceptual categories, and subcategories and asked to match the meaning units and their associated open codes to the correct categories. The auditor was able to correctly allocate 49 of the 50 open codes to their associated categories. On only one occasion did he struggle to allocate a meaning unit to the correct category, and he attributed this to being unfamiliar with CF.

## 3. Results

### 3.1. Sociodemographic Details

Between them the 11 participants (aged 22–41 years) had 13 children (aged seven months to 14 years), and one was also six months pregnant with her second child. Ten children were conceived “naturally,” two were conceived via intrauterine insemination (IUI), one was conceived via in vitro fertilisation (IVF), and one child was adopted. Four participants had completed undergraduate degrees, and seven had studied to A-Level and the others to GCSE. Two participants did not provide this information. Seven participants were homemakers, three worked part time, and one worked full time (paid). Ten were married and one was cohabiting. All partners were male and reported to be in full-time employment.

### 3.2. Quantitative Measures

Results from the HADS [29] and CFQoL Scale [30] revealed no outstanding difficulties with emotional functioning and/or quality of life and were comparable to results from the general female CF population [33, 34].

### 3.3. Qualitative Data

#### 3.3.1. Theoretical Formulation

**Figure 1:**
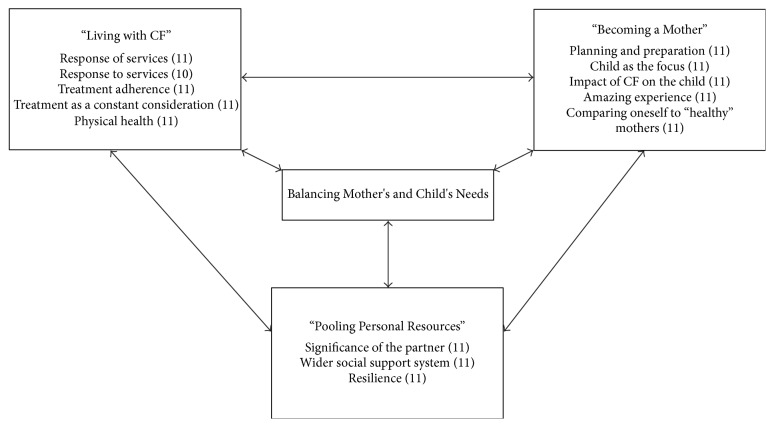
Theoretical formulation: the relationships between the three core categories around a central process and the conceptual categories contained within them. Numbers in brackets indicate number of participants contributing to that category.

The theoretical formulation details the three core categories (and the conceptual categories contained within them) and how they relate via a central process (Figure 1).

The first core category, “Living with CF,” relates to the day-to-day issues involved in living with the condition, regardless of parenthood. The second, “Becoming a Mother,” relates specifically to issues associated with having children. These core categories mutually influence one another and involve two sets of needs that have to be balanced, such as that of the mother's for rest and treatment and the child's for care and attention. A central way that participants achieved this balance was by “Pooling Personal Resources”—the third core category. All three core categories are linked through a central process: “Balancing Mother's and Child's Needs.”

## 4. Core Category One: “Living with CF”

The first core category described the day-to-day management of CF and contained five conceptual categories: response of services, response to services, treatment adherence, treatment as a constant consideration, and physical health.

### 4.1. Response of Services (CF Teams' Response to the Desire for, and Achievement of, Parenthood)

There was a theme of variation in perceived support for the mothers' desires and decisions to have children by their CF team. The majority of participants felt unsupported in their decision at some point, although many conceptualised this as caution by the team based on their health status at the time. Others described inconsistencies between and within CF teams which they felt was not always linked to health status. Around half the participants felt their CF team lacked experience in managing pregnancy, leading to provision of little or inaccurate information and lack of appropriate care. Some felt the communication between their CF and obstetric teams was inadequate and problematic.

Participants discussed what care they wanted from their team, including increased contact following the birth and more emotional support:

*“I definitely think that there could have been more emotional support, even just contact and a bit of “how are you?” type thing.”* (Pippa)

The majority felt that conversations with their team around their “day-to-day” life as a mother would help with treatment planning and in nurturing their relationships with them. They also discussed the importance of CF teams acknowledging the practicalities and difficulties of balancing treatment with parenting, particularly in arranging appointment times, in supporting them to do intravenous treatment at home where possible, and in developing alternative treatment regimens:

*“They were really good at coming up with new techniques and ways that I could do [physiotherapy] while I was looking after Ava…and the same with the nebuliser as well…they gave me breathing techniques so that I could still do that physio.”* (Rachel)

### 4.2. Response to Services (Participants' Responses to CF Team Support)

In general, the CF team were a valued source of support and information. The majority of participants had sought the opinions of their teams before making any firm decisions about motherhood. There was a tendency to give weight to doctors' opinion, although some thought this was not always up to date or accurate and would sometimes challenge it:

*“There was no way that I would have got pregnant without the support of my team…I had every faith that they're looking after me in the best possible way.”* (Rachel)

*“I was just like-I couldn't care what you say anyway, I'm doing to do what I want.”* (Adele)

Some participants described reducing their contact with their team or a particular doctor due to a lack of support of their decision to have children, which had an impact on their CF care:

*“Say if I wasn't well or something, I wouldn't phone them straight away…I didn't want to see them.”* (Adele)

### 4.3. Treatment Adherence (Perceived Impact of Motherhood on Adherence to Treatment and How It Changed over Time)

The majority of participants described an initial disruption to treatment on becoming a mother, with some or all of their treatment getting missed (particularly physiotherapy and nebulisers). All but one participant described reestablishing their full treatment regime, usually within 2-3 months. Participants cited a lack of time for themselves as being a reason for treatment disruption, as well as disruption to their treatment routine. All discussed the importance of reestablishing new treatment routines and noted that this became easier over time, as they developed routines with their children:

*“You have to organise yourself and kind of work out a routine so that it fits in for you and your little one.”* (Rachel)

Later, still, increased independence and understanding in their children led to participants having more time and thus increased adherence. All felt they were more adherent to their treatment after having children, due to their need and desire to stay well for them both in the short and in the long term:

*“I used to be quite lax-a-daisy in terms of doing physio, but since having Noah I would say the treatment and physio have definitely increased...you know someone else needs you.”* (Heidi)

### 4.4. Treatment as a Constant Consideration (the Continuing, and Increasing, Importance of Treatment to the Participants)

The majority of participants acknowledged that CF treatment had become even more of a priority for them. Many invested in adapting treatment times and routines, placing themselves under pressure to maintain these and feeling upset if they missed them:

*“[treatment] became even more of a priority because I have to be here to look after Henry.” *(Jessica)

*“I had to take care of myself because I didn't want to be sick, I wanted to spend time with him, and be able to run around and play with him and stuff.”* (Chloe)

### 4.5. Physical Health (Interaction between Health and Mothering)

Some participants noted deterioration in their health after having children, with most attributing this to disruption in treatment. The majority discussed the impact their health sometimes had on mothering activities, for example, sometimes having to limit engagement in exhausting activities:

*“She loves rough and tumble play, being thrown up in the air…that sort of thing I find that harder…so Izzy and I, we'll do lots of crafty things instead.”* (Joanna)

Similarly, mothering was also noted to affect their health (e.g., feeling fatigued). Fluctuating health and noting limited health as impacting mothering activities caused participants upset and frustration and guilt if they felt their CF was impacting on their parenting:

*“I feel a bit more limited in my energy…so there's sort of a daily guilt…that I'm not providing as fuller life for him than I would do if I didn't have CF.”* (Amy)

Adequate time for rest was highlighted as important in managing this.

## 5. Core Category Two: “Becoming a Mother”

The second core category described issues specifically associated with motherhood and contained five conceptual categories: planning and preparation, child as the focus, impact of CF on the child, amazing experience, and comparing oneself to healthy mothers.

### 5.1. Planning and Preparation (the Planning and Preparation Which Participants Associated with Becoming a Mother)

All expressed long-standing desires to have children, referring to the amount of thought that had gone in to their decisions. Due to perceptions of limited life expectancy, many felt they had begun considering children earlier than they would have done had they not had CF; however half thought that it would never be possible. The majority of participants attempted to prepare themselves physically for motherhood (e.g., increasing lung function and putting on weight). However, preparation for other aspects was more problematic, given they felt there was a lack of psychosocial information given to them by their team relating to CF and motherhood. Some also felt there had been too much focus on pregnancy during discussions with their CF team and not enough on what life as a mother may be like:

*“A lot of the doctors go well yes you can get through a pregnancy and they don't begin to talk about, plan can you be a mum, can you look after a child.”* (Tamsin)

*“I always wanted to know at the time…a mum with CF…what does their day to day life look like and what's different from them being a mum…it felt very impersonal I think when we talked about it, the decision to start a family, with my team, I felt they couldn't offer me a lot of the ‘experience' stuff, which is what I wanted to hear.”* (Amy)

Participants emphasised the importance of being organised and planning ahead in order to manage the dual demands of CF and motherhood.

### 5.2. Child as the Focus (Impact and Importance of the Child)

All participants discussed the amount of attention that children, particularly babies, required, and this was given as a major cause of treatment disruption, particularly during the first few months:

*“For the first few weeks all I seemed to do was feed and express and everything (treatment) went out of the window.”* (Jessica)

The majority of participants discussed the sense of responsibility they felt on becoming a mother and a subsequent pressure to stay well. Participants worked hard to stay well, in order to keep themselves well to be able to play, avoid hospital, and extend their life expectancy:

*“I absolutely to the letter do everything. I'd say that's purely as a result of having a child…it's the awareness you are now responsible for someone else, you owe it to them to keep yourself in good shape.”* (Amy)

All had adapted their treatment routine to fit around their children's, which appeared helpful in facilitating adherence:

*“It's about making a new [routine] that focuses around the kids completely, and then the CF part fits into the kids.”* (Adele)

### 5.3. Impact of CF on the Child (Impact that the Mothers' CF May Have on the Experience of the Child)

The majority of participants acknowledged the possibility of having a shortened life expectancy, which often led to feelings of guilt, as did having time away from their children for hospital admissions and treatment:

*“I really do feel guilty sometimes, and I think oh gosh I'm like putting my son through that, you know what's to come sort of in the future.”* (Kimberley)

*“I was in hospital for two weeks…I found that tremendously hard…you just feel really guilty that you're not there.”* (Joanna)

They reflected too on the impact of their children witnessing their CF treatment, which was felt to be positive, helping to “normalise” CF and increase their children's understanding of it. Participants noted their children's frustration at sometimes not having their mother's full attention; however some actively involved their children in part of their treatment regime and felt this facilitated adherence, minimised children's frustration at lack of attention, and increased their understanding:

*“He even helps me get some of it ready…I make [treatment] in to a game rather than make it into something morbid.”* (Adele)

Participants discussed how they wanted their children to understand CF in order to minimise any “shock” later on but described this as being difficult, particularly with younger children:

*“Trying to explain it in a way that's not too scary for him…but in a way that still makes him understand…it's quite tricky.”* (Amy)

The majority of participants focused on explaining the short-term implications of CF, such as the need for treatment, rather than the longer term implications.

### 5.4. Amazing Experience

Enjoyment and disbelief at becoming a parent were common reflections, with some participants feeling a great sense of achievement. No mother expressed regrets over having had children, and instead they discussed the joy that their children brought them, in addition to the motivation to stay well and adhere to treatment:

*“They bring so much joy to your life and it gives me a reason to try harder, I want to be around longer.”* (Kimberley)

### 5.5. Comparing Oneself to Healthy Mothers

This category contained references to perceived similarities and differences to healthy mothers. The majority of participants discussed feeling different to mothers without CF or another health condition, describing feeling that being a mother with CF is harder given the added burden of treatment and increased fatigue:

*“I've had many a conversation with normal mums who say they're tired, and I think, yes so am I, but I also have to do X, Y and Z treatment!”* (Ellie)

However, all participants also saw similarities in the changes and issues they had experienced to their perceived experiences of “healthy” parents.

## 6. Core Category Three: “Pooling Personal Resources”

The third core category described the use of social support in coping with the new demands of being a mother. It contained three conceptual categories: significance of the partner, wider support system, and resilience.

### 6.1. Significance of the Partner (Significance Ascribed to the Partner)

The majority of participants thought their partner had been their most important source of support in coping with motherhood and in balancing this with CF. Partners support allowed time out to rest and do treatment:

*“For a good few months I had to rely on my other half to take her off me, just to have half an hour so I could sit and do some treatments.”* (Pippa)

Partners were also described as being important in helping to manage the impact CF sometimes has on mothering:

*“He can do things like lift, when I'm not well, he can lift the kids in and out of the bath, he can carry the pram in and out of the car…if I was single and on my own I would really struggle.”* (Adele)

Some participants also described receiving emotional support from their partner and the majority also acknowledged the significant impact becoming a parent had had on him.

### 6.2. Wider Social Support System (Other Sources of Support)

The majority of participants acknowledged how significant the support of their friends and wider family had been in coping with motherhood. This enabled participants to care for themselves and was particularly important when their partner was unavailable or when they felt unwell:

*“When I've been at home and not felt very well it's a chance for mum and dad, they take Izzy out…I know she's having a lovely time…you're not then worrying that you not feeling very well is impacting more than it has to on her.”* (Joanna)

*“I've got a lot of support and that's a major major thing to have that support, otherwise I'd not have had any chance of doing it.”* (Kimberley)

Support often involved practical support (e.g., cooking). Some felt having children had brought their family closer together, often consciously due to an awareness of their limited life expectancy.

### 6.3. Resilience (Successful Personal Coping Strategies)

Participants drew on their own personal resilience. They discussed how they had adopted an attitude of “just getting on with it” in coping with the complex demands of CF and motherhood:

*“You just get on with it…you get out and you just get on with it.”* (Tamsin)

Participants also discussed drawing on their own personal strength of being able to think positively and focusing on the present. Some described a process of “working out” their own way of coping with the demands of motherhood:

*“At the beginning with a newborn, you're new to it…you're learning at the same time…and now he's getting older…it's just a constant learning curve.”* (Chloe)

### 6.4. Central Process: “Balancing Mother's and Child's Needs”

Finally, a central process was highlighted that referred to the way participants strove to balance the twin demands of having CF and being a mother. They discussed how, in the early stages of becoming a mother, it was difficult to balance caring for their children with their need for treatment and rest, and their treatment routine could be disrupted as a result. They often had to put their own needs above their child's, leading to feelings of guilt. However, there was an awareness described by all that prioritising their own health needs was also ultimately also for the well-being of their children:

*“You almost have to be a little bit selfish and say to yourself that, that's maybe the approach I have taken, that I'm no good to Izzy if I'm poorly, and sometimes that will mean that, physio is a perfect example I think, she might not like it, but I think you've just got to do it.”* (Joanna)

Participants worked hard to achieve this balance and described using a number of strategies, including utilising the support offered by partners and extended family, fitting their treatment around their children's routines, and involving their children in treatment. There was recognition that the ability to find this balance became easier over time as their children became more independent, they had more time to themselves, and they became more adept at establishing and maintaining routines.

## 7. Discussion

Participants described many similar experiences to those described by healthy mothers, such as feelings of joy, a huge sense of responsibility, fatigue, and a lack of time to themselves [4–6, 9]. However, there was no mention of other themes commonly discussed by healthy mothers such as feelings of loss, isolation, and perceptions of incompetence as mothers [4, 6, 7].

Participants discussed a number of emotional experiences similar to those described by mothers with other chronic health conditions, including feelings of guilt and anxiety associated with feeling unwell, limited energy, and reduced life expectancy [11, 12, 14]. Participants also discussed how poor health led to feeling upset and frustrated, as did observing the impact their health had on mothering activities and vice versa, as discussed by other chronically ill mothers [11, 20].

In this study, participants reflected on the impact that having a mother with CF may have on their children, with an awareness of their children's frustration at not having their full attention at times (due to treatment). Overall, participants felt it was helpful for their children to witness their treatment regimens and to have a good understanding of their condition (which witnessing treatment was felt to help), in order to minimise any negative impact such as feelings of “shock” later on and to “normalise” their treatment and health limitations. This is in contrast to reports from some mothers with other chronic conditions who have reported feeling it best to “hide” their illness and/or treatment in order to protect their children [16]. However, given it has been mainly studies with mothers with HIV where avoidance of disclosure has been discussed, the different findings may be due to CF being different to HIV in regard to the stigma attached. It must also be noted that participants in this study focused on explaining the “short-term” implications of CF (e.g., the need for treatment), rather than longer term ones, such as the potential for reduced life expectancy. This may be due to the young age of the majority of their children.

Further differences were also noted when compared to reports from other chronically ill mothers. Participants in this study did not appear to feel any insecurity that they may not meet “societal expectations” of the “ideal” mother and placed no pressure on themselves to achieve it, as described by other chronically ill mothers [18]. Neither did they express concerns they could not protect their children, as discussed by other chronically ill mothers [12, 20]. This is commensurate with other parents with CF who did not report to believe that having CF prevented them from being good parents [35].

While disruption to CF care was expressed by participants, they also noted that it became easier to adhere to treatment over time. Reasons given for this included increased time for treatment due to establishing routines and children's increasing independence and understanding. All participants described an overall improvement in adherence rates when compared to their rates of adherence prior to having children, due to a desire and sense of responsibility to stay well, reflecting experiences of mothers with other chronic conditions [11, 15, 20]. Participants described wanting to stay well so that they could play with their children and avoid hospital admissions and to extend their life expectancy as much as possible.

Participants discussed a central process of needing to constantly balance their own needs for rest and treatment with their children's needs for care and attention. This balance became easier to achieve over time due to the reasons given for increased adherence. However, participants also drew on a range of coping strategies in helping them to achieve this balance. One such coping strategy included utilising social support. Partners were identified as the most significant source of support, mirroring findings of mothers with and without other chronic health conditions [6, 8, 10, 20]. Some stated the importance of the support of extended family. Most also drew on their own resilience. Other proactive strategies in coping and adjusting to motherhood and in attempting to find this balance included adapting treatment routines to fit around their children, swapping exhausting activities for less draining ones (also discussed by mothers with multiple sclerosis and asthma) [12, 18], planning ahead, and being organised. Some even involved their children in treatment. This latter strategy does not appear to have been discussed by mothers with other chronic conditions; however professionals in the area of CF have previously suggested it may be helpful in increasing adherence and children's understanding [36]. This certainly appeared to be the case in the present study.

Overall, participants appeared to be coping and well-adjusted to motherhood. This may be due to them engaging in a number of processes thought to be necessary for effective adjustment such as “assigning meaning” (e.g., reflecting on reasons for health deterioration) [37] and “a search for mastery” (e.g., thinking positively, seeking information, and complying with treatment) [38], in addition to the use of coping strategies that fit appropriately with the controllability of the situation [39]. Furthermore, participants and their families demonstrated a number of processes thought to be significant for family adjustment, such as the ability to organise themselves effectively [40]. Participants also all had extensive support systems.

The majority of doctors were supportive of participants' decisions to have children; however when this was not overt some participants “withdrew” from their teams. Participants noted the desire for psychosocial information relating to motherhood prior to, and during, pregnancy, but a significant lack of this and focus on medical issues. The need for psychosocial information relating to chronic illness and motherhood is well-established [10, 25]; however it is generally agreed that such information is lacking. The present study appeared to suggest this.

This study was rigorous in its application of GT (e.g., adopting a number of strategies to ensure that the conclusions were credible and “grounding” the data gathered from the participants). Recruitment from the online forum CFMothers.com offered the advantage of obtaining a UK-wide sample. However, like all opt-in samples, it could be argued that these participants were not representative of the wider group of CF mothers in that they took the initiative to seek out other mothers for support. It might also be argued that they were “atypical” in their high levels of reported adherence, though their own explanation of this was that it reflected increased motivation after parenthood. The participants in this study were certainly generally in good health, were financially comfortable, had supportive partners and many did not work outside the home. A majority also described effective and close support systems, the presence of which has been found to enhance adjustment to motherhood and to increase adherence to treatment in chronically ill mothers [12, 40]. On the other hand, participants seem representative of adult CF patients in terms of their physical health and emotional functioning and quality of life as measured by the HADS and CFQoL Scale.

Future research might extend the sample to include those in poorer health or, as the children of the mothers in the current study were comparatively young, track the adaptations and changing needs of mothers over time. It is also important to understand the experience of fathers with CF to inform appropriate care, given the advancements in assisted reproductive technology.

## 8. Conclusions

A number of complex and interrelating psychosocial processes are experienced by women with CF when they have children and a range of coping and adjustment processes take place as a result.

The theory developed in this study presents a rich account of the impact of motherhood and highlights relationships among the processes experienced. A number of novel findings were evident, suggesting that (i) complete treatment disruption is not inevitable with some participants experiencing little disruption and disruption being broadly time-limited, (ii) the utilisation of social support is not the only way of coping with good evidence of other forms of “problem-solving” and displaying of resilience, (iii) “emotional burden” is not always present over disclosing their CF to their child, (iv) having the CF team support decisions about pregnancy and parenthood is vital, and this needs be overtly conveyed, and (v) CF teams need a detailed understanding of motherhood and CF in order to provide information during discussions about having children and when planning for motherhood.

## 9. Practice Implications

Recommendations for clinical practice based on the results are outlined in Table 2.

## Figures and Tables

**Table 1:** Health and treatment status of participants (“—” indicates missing data).

Participant	Age at diagnosis (in years)	FEV_1 _%	BMI	Chest Clearance Physio.	Nebulised treatment	Pancreatic enzymes	Vitamins	Inhalers	Antibiotics (oral)	CF-related diabetes
Pippa	6	68	27	✓	✓	✓	✓	✓	✓	✓
Tamsin	0.25	78	23	✓	✓	✓	✓	✓	✓	*✘*
Ellie	0.5	67	20	*✘*	✓	✓	✓	✓	✓	*✘*
Jessica	2	77	31	✓	✓	*✘*	*✘*	*✘*	✓	✓
Chloe	20	—	—	✓	✓	—	—	—	—	—
Kimberley	14	42	21	*✘*	✓	*✘*	*✘*	✓	✓	*✘*
Amy	1.5	77	22	✓	✓	✓	✓	*✘*	✓	✓
Joanna	4	27	21	✓	✓	✓	✓	*✘*	✓	✓
Heidi	Birth	75	21	✓	✓	✓	✓	✓	✓	*✘*
Rachel	0.25	68	22	✓	✓	✓	✓	*✘*	✓	*✘*
Adele	0.75	103	—	*✘*	✓	✓	✓	—	✓	✓

**Table 2:** Recommendations for clinical practice.

Recommendation	Detail
Relationship with the CF team	It is important that CF teams work to build and maintain effective relationships with their patients, being as supportive as possible. General “lifestyle” discussions can facilitate this
Opening up discussions	It is important that CF teams open up discussions about motherhood as early as it is felt to be appropriate
Information giving	Prior to, and during, the planning and preparing for motherhood, female patients should be given psychosocial information relating to motherhood as well as information on physical risks and health implications, in order to help them plan and prepare and to increase the accuracy of their expectations. The results of this research can serve as a useful foundation for information to be delivered to female patients
Needs assessment	A reassessment of the new needs of the mother should be undertaken to establish these and how they may best be met
Managing health and treatment	It is important that CF teams remain consistently aware of, and understand, a mother's dual commitments and help manage these wherever possible to relive the burdens of CF-related care as much as possible
Facilitating support	It is important that CF teams help to facilitate access to support for the new mother. This may include practical and emotional support from an appropriate team member. It is also important that the needs of her partner are also taken into consideration
CFMothers.com	Female patients with CF may benefit from being directed to the CFMothers.com website
Communication and education	It is important that CF teams liaise closely with obstetric teams throughout the pregnancy and that obstetric teams have a good understanding of CF
Following guidelines	It is important that female patients are given consistent and accurate information in regard to pregnancy and motherhood, to reduce any distress and confusion
